# Antiphotoaging Effects of 3,5-Dicaffeoyl-epi-quinic Acid via Inhibition of Matrix Metalloproteinases in UVB-Irradiated Human Keratinocytes

**DOI:** 10.1155/2020/8949272

**Published:** 2020-04-28

**Authors:** Jung Hwan Oh, Jung Im Lee, Fatih Karadeniz, So Young Park, Youngwan Seo, Chang-Suk Kong

**Affiliations:** ^1^Marine Biotechnology Center for Pharmaceuticals and Foods, College of Medical and Life Sciences, Silla University, Busan 46958, Republic of Korea; ^2^Department of Food and Nutrition, College of Medical and Life Sciences, Silla University, Busan 46958, Republic of Korea; ^3^Division of Marine Bioscience, Korea Maritime and Ocean University, Busan 49112, Republic of Korea

## Abstract

UVB exposure is one of the causes of several skin complications including but not limited to premature aging, wrinkle formation, and hyperpigmentation. UV-induced skin aging is called photoaging, and oxidative stress-induced overexpression of matrix metalloproteinases (MMPs) is the main reason behind the photoaging-mediated collagen degradation. Natural origin inhibitors of MMPs are regarded as a promising approach to prevent or treat photoaging. Therefore, the present study investigated the protective effects of 3,5-dicaffeoyl-epi-quinic acid (DCEQA) in human HaCaT keratinocytes against UVB irradiation-related dysregulation of MMPs. Changes in the mRNA and protein expression and release of MMP-1, -2, and -9 were observed after UVB irradiation with or without DCEQA treatment. In addition, the effect of DCEQA on the activation of p38, JNK, and ERK MAPKs was analyzed. Treatment of UVB-irradiated HaCaT cells with 10 *μ*M DCEQA significantly suppressed the overexpression of both mRNA and protein of MMP-1, -2, and -9 while slightly increasing the diminished type I procollagen production. UVB-induced activation of MAPKs was also ameliorated by DCEQA treatment in a dose-dependent manner. Results indicated that DCEQA treatment was able to protect keratinocytes from UVB-induced photoaging by inhibiting the stimulated production of MMPs and the related decrease in collagen production. It was suggested that DCEQA downregulated the collagen degradation via inhibition of MAPK activation, which resulted in decreased MMP activity.

## 1. Introduction

The structure, function, and appearance of human skin are being threatened constantly by several hazardous environmental factors. Ultraviolet (UV) irradiation is one of those factors that has notable detrimental effects on the skin including cancer, sunburn, inflammation, and photoaging [1]. Studies have suggested various pathways through which UV irradiation implements its harmful effects on skin structure and function. Among all the deteriorations caused by UV, photoaging is considered to progress via damaged extracellular matrix (ECM) which is the connecting tissue in the skin layers [2, 3]. It has been demonstrated that photoaging skin comprises modified ECM structures, especially in the collagenous backbone [4]. Matrix metalloproteinases (MMPs) are endopeptidases that are responsible for the ECM modification and remodeling of the collagenous layers via digestion of the collagen [5]. Collagen is the main component of the extracellular matrix and connective tissues and is credited to be the crucial part of the strong and elastic skin. It is synthesized by dermis fibroblasts, and the leading characteristic of the photodamage in the skin is fragmented, damaged, or disorganized collagenous backbone [3].

Ultraviolet B (UVB) is one of the light rays that are emitted by the sun together with UVA and UVC. UVB irradiation was shown to notably increase the production of reactive oxygen species (ROS) in the skin after a short time following the exposure [6]. The link between UVB exposure and photoaging was suggested to be the cleavage of collagen [7]. UVB-induced elevated generation of ROS stimulates the expression and enzymatic activity of MMPs, especially MMP-1, -2, and -9, which in turn causes the collagen to be rapidly digested [8, 9]. MMPs are crucially involved in the remodeling of ECM, migration of cells, and photoaging. This cascade of stimulation results in the damaged ECM, the reason for the formation of wrinkles, and deformed, prematurely aged skin [10]. Keratinocytes are the main cell type of the epidermis and are responsible for forming a barrier against extrinsic factors such as UV radiation, bacteria, virus, and parasites. UVB rays can penetrate the skin until the basal layers of the epidermis and exert harmful effects on keratinocytes [4]. UVB-irradiated keratinocytes were shown to overexpress MMPs via ROS-mediated MAPK activation. Overproduction of MMPs in keratinocytes exposed to harmful doses of UVB results in diminished collagen production and increased cleavage of ECM components such as collagen and elastin.

Considering the importance of MMPs in harmful effects of UV irradiation in the skin as well as in other diseases and complications such as tumor metastasis, inflammation, atherosclerosis, and cardiovascular diseases, numerous effective MMP inhibitors have been studied and developed. The considerable portion of MMP inhibitors was derived from natural sources, mainly phytochemicals such as polyphenols, flavonoids, coumarins, caffeic acids, and their derivatives [11–13]. Caffeic acids are naturally occurring phenolic compounds found widespread in plant products such as vegetables, fruits, oils, wines, and coffee along with agricultural products such as propolis [14]. Past decades researchers reported various bioactivities of caffeoylquinic acid (CQA), a derivative of caffeic acid [15]. Up to date, CQA and CQA-based compounds have been exhibited to show important health benefits such as antioxidant [16], antihistaminic [17], antibacterial [18], antiviral [19], and anticancer [20] activities. As a part of our continuous effort to develop natural products with bioactivities, 3,5-dicaffeoyl-epi-quinic acid (DCEQA), a bioactive derivative of CQA, has been shown to possess antiobesity [21] and photoprotective antioxidant abilities [22]. The current study is aimed at evaluating the photoprotective effect of DCEQA against UVB-induced changes in skin cells and analyzing its possible mechanism of action for antiphotoaging potential.

## 2. Materials and Methods

### 2.1. Isolation of 3,5-Dicaffeoyl-epi-quinic Acid

3,5-Dicaffeoyl-epi-quinic acid (DCEQA) was isolated from *Atriplex gmelinii* as a white powder. Isolation, characterization, and identification of the compound were carried out as reported earlier [21].

### 2.2. HaCaT Human Keratinocyte Culture and Cytotoxicity Assay

HaCaT cells (300493; Cell Line Service, Eppelheim, Germany) were cultured in Dulbecco's modified Eagle medium with 10% fetal bovine serum (FBS), and cells were kept in 37°C incubators with an atmosphere containing 5% CO_2_ between the experiments.

Any possible toxic effect of DCEQA in cells was investigated by colorimetric MTT assay as previously described (Bae et al., 2016). Briefly, HaCaT keratinocytes were seeded in 96-well plates and treated with 1, 5, and 10 *μ*M DCEQA along with untreated control group. The culture medium was replaced with 100 *μ*l MTT solution (1 mg/mL) following a 2-day incubation. The plate was then kept in dark at 37°C for 4 h. Wells were aspirated, and cells were washed with phosphate buffer saline (PBS). Ten microliters of 100% DMSO were introduced to each well to dissolve the formazan crystals, and absorbance values of wells at 540 nm were measured using GENios FL microplate reader (Tecan Austria GmbH, Grodig, Austria). Changes in the viability of cells were calculated as a percentage of the untreated blank group which was considered to be 100% alive and compared to each concentration of DCEQA treatment.

### 2.3. Enzyme-Linked Immunosorbent Assay (ELISA)

Release of MMP-1, -2, and -9 and type Iα1 procollagen from UVB-irradiated HaCaT cells was analyzed by ELISA. Cells were preincubated in 6-well plates for 24 h and washed with PBS prior to UVB (15 mJ/cm^2^) exposure. After UVB irradiation, the cells were treated with or without different concentrations (1, 5, 10 *μ*M) of DCEQA for 24 h. Cell culture medium from each well was analyzed for its MMP-1, -2, and -9 and type I*α*1 procollagen contents as per manufacturer's instructions of the ELISA kit (R & D Systems, Inc., Minneapolis, MN, USA).

### 2.4. Reverse Transcription Semiquantitative Polymerase Chain Reaction (RT-PCR) Analysis

Total RNA was isolated from nonirradiated and irradiated (UVB, 15 mJ/cm^2^) HaCaT keratinocytes using TRIzol® reagent (Thermo Fisher Scientific, Rockford, IL, USA). RNA (2 *μ*g) and oligo (dT) were mixed in RNase-free water for the cDNA synthesis. The synthesis was performed in a thermocycler (T100; Bio-Rad Laboratories, Inc., Hercules, CA, USA) with an initial denaturation of the mix at 70°C for 5 min and immediate cooling, followed by the preparation of a master mix containing 1X RT buffer, 1 mM dNTPs, 500 ng oligo (dT), 140-unit M-MLV reserve transcriptase, and 40-unit RNase inhibitor. The remaining cycles consisted of 42°C for 60 min and 72°C for 5 min. The target cDNA was amplified using the sense and antisense primers as previously noted [21]. The amplification was carried out with 30 cycles; each cycle consisted of 95°C for 45 sec, 60°C for 1 min, and 72°C for 45 sec. The final PCR products were separated by agarose gel (1.5%) electrophoresis for 30 min at 100 V. Gels were then stained with 1 mg/ml ethidium bromide and visualized by UV light using Davinch-Chemi imager™ (CAS-400SM, Seoul, Korea). Quantification was carried out by band density measurement which was analyzed using MultiGauge software (v. 3.0; Fujifilm, Tokyo, Japan).

### 2.5. Western Blotting

Protein levels were investigated using immunoblotting according to common Western blotting protocols. HaCaT cells cultured in 6-well plates were treated with or without DCEQA (1, 5, 10 *μ*M) for 24 h after UVB (15 mJ/cm^2^) irradiation. Following incubation, wells were aspirated and cells were lysed by vigorous pipetting in 1 ml of RIPA buffer (Sigma-Aldrich, St. Louis, MO, USA) at 4°C. The protein content of the lysates was measured with a BCA protein assay kit (Thermo Fisher Scientific) following the kit's protocol. The same amount (20 *μ*g) of protein from each well was loaded onto 12% SDS-polyacrylamide gel and run at 100 V. Proteins on the gel were then transferred onto a polyvinylidene fluoride membrane (Amersham; GE Healthcare, Little Chalfont, UK) using a wet system run at 100 V for 1 h at 4°C. Membranes were then incubated for 1 h at room temperature in 5% skimmed milk for blocking. Blocked membranes were washed with 1X TBST and incubated with primary antibodies against MMP-1 (sc-6837; Santa Cruz Biotechnology, Santa Cruz, CA, USA), MMP-2 (#4022; Cell Signaling Technology, Beverly, MA, USA), MMP-9 (#393857; Cell Signaling Technology), type I procollagen (sc-8782; Santa Cruz Biotechnology), p38 (#8690; Cell Signaling Technology), phospho (p)-p38 (#4511; Cell Signaling Technology), JNK (LF-PA0047; Thermo Fisher Scientific), p-JNK (sc-293136; Santa Cruz Biotechnology), ERK (#4695; Cell Signaling Technology), p-ERK (#4370; Cell Signaling Technology), and *β*-actin (sc-47778; Santa Cruz Biotechnology) in primary antibody dilution buffer containing 1X TBST with 5% bovine serum albumin, overnight at 4°C. Membranes were then incubated with horseradish-peroxidase-conjugated secondary antibodies (diluted 1 : 1,000) specific to the primary antibody source organism at room temperature for 1 h. Detection of proteins on blotted membranes was achieved using an ECL Western blot detection kit (Amersham) according to the manufacturer's instructions. Protein bands were imaged with CAS-400SM Davinch-Chemi imager™ (Davinch-K) and bands were quantified from images densitometrically.

### 2.6. Flow Cytometry

The MAPK (ERK1/2) activation levels were investigated employing flow cytometry. Cells were preincubated in 6-well plates (1 × 10^6^ cell/well) for 24 h and washed with PBS prior to UVB (15 mJ/cm^2^) exposure. Following UVB irradiation, the cells were treated with or without DCEQA (10 *μ*M) for 24 h. Levels of ERK1/2 phosphorylation were measured with MUSE^TM^ MAPK Activation Dual Detection kit (MCH200104; Merck KGaA, Darmstadt, Germany) using MUSE^TM^ Cell Analyzer software (Muse Cell Soft v. 1.4.0.0, Merck KGaA, Darmstadt, Germany) according to the manufacturer's instructions.

### 2.7. Statistical Analysis

All numerical data were given as the mean ± standard deviation of three separate experiments carried out in triplicate. Statistical differences between the means of the sample groups were calculated by the analysis of variance (ANOVA) followed by Duncan's multiple range test using SAS v. 9.1 software (SAS Institute, Cary, NC, USA). Any statistically significant difference between the groups was determined at *p* < 0.05 level.

## 3. Results

### 3.1. Cytotoxicity of UVB Irradiation and DCEQA Treatment in HaCaT Cells

Human immortalized HaCaT keratinocyte cell line was used as an in vitro model for the assays. These cells produce elevated levels of reactive oxygen species (ROS) and overexpress MMP-1, -2, and -9 when exposed to UV irradiation.

**Figure 1:**
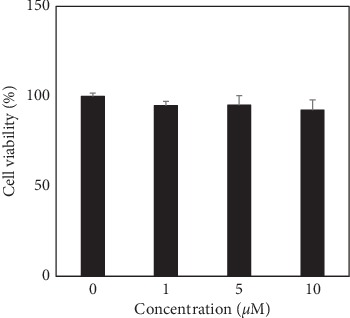
Effect of DCEQA on the viability of HaCaT human keratinocytes analyzed by MTT assay. The viability of the cells after 24 h treatment with DCEQA at the indicated concentrations was calculated and given as the percentage of untreated control cells (0 *μ*M).

Prior to elucidating the potential photoprotective effects of DCEQA against UVB irradiation, its biocompatibility was assessed by MTT cell viability assay in HaCaT human keratinocytes. Treatment with varying concentrations (0, 1, 5, and 10 *μ*M) of DCEQA for 48 h did not show any cytotoxic effect in HaCaT cells (Figure 1).

**Figure 2:**
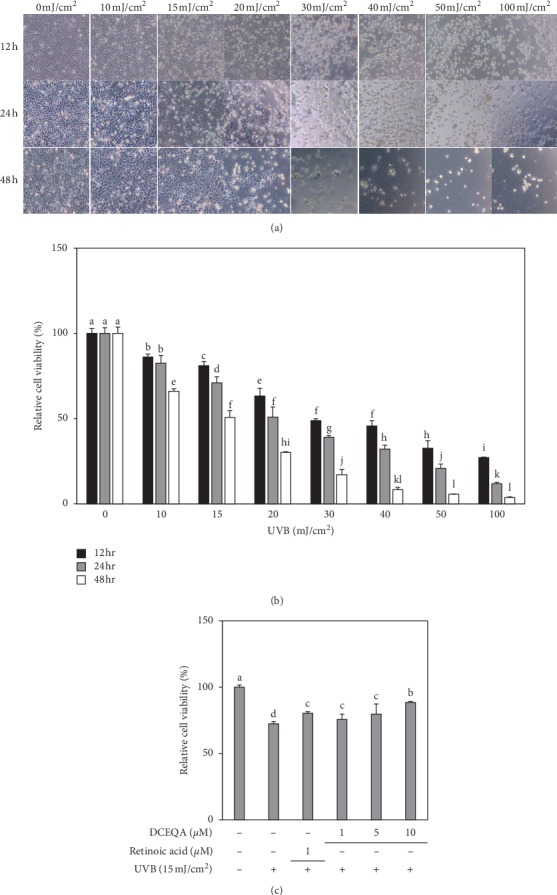
Images and viability of HaCaT cells irradiated by different doses (0–100 mJ/cm^2^) of UVB radiation and incubated for 12, 24, and 48 h (a). Relative cell proliferation of HaCaT cells treated with DCEQA (1, 5, 10 *μ*M) after UVB (15 mJ/cm^2^) irradiation and incubated for 24 h (b). Viability of the cells after UVB irradiation and treatment with DCEQA was investigated by MTT assay and given as the percentage of nonirradiated or untreated (0 *μ*M) control cells. ^a-d^Means with different letters are significantly different at *p* < 0.05 level.

In order to determine the dose of UVB radiation exposure, HaCaT cells were exposed to a range of UVB doses (0–100 mJ/cm^2^) and incubated for 12, 24, and 48 h. Comparing the relative cell viability and cell morphologies, we chose 15 mJ/cm^2^ exposure as the minimal dose to induce a 50% loss in cell viability (Figure 2(a)). At these conditions, almost half of the HaCaT cells were not viable while the morphology of the live cells was similar to unirradiated control cells.

### 3.2. Cytoprotective Effect of DCEQA against UVB-Induced Cytotoxicity

HaCaT cells were exposed to UVB radiation (15 mJ/cm^2^) and treated with different concentrations of DCEQA for 24 h. Treatment with DCEQA protected the cells from UVB-induced suppression of proliferation as the live cells were higher than the untreated irradiated control group (Figure 2(b)). UVB exposure caused a 27.55% decrease in untreated cells compared to nonirradiated untreated blank cells. Treatment with 10 *μ*M DCEQA resulted in an 11.54% decrease, while positive control retinoic acid (1 *μ*M) was able to keep the decrease at 19.72%.

### 3.3. Effect of DCEQA on UVB-Induced Secretion and Expression of MMPs and Type Iα1 Procollagen

**Figure 3:**
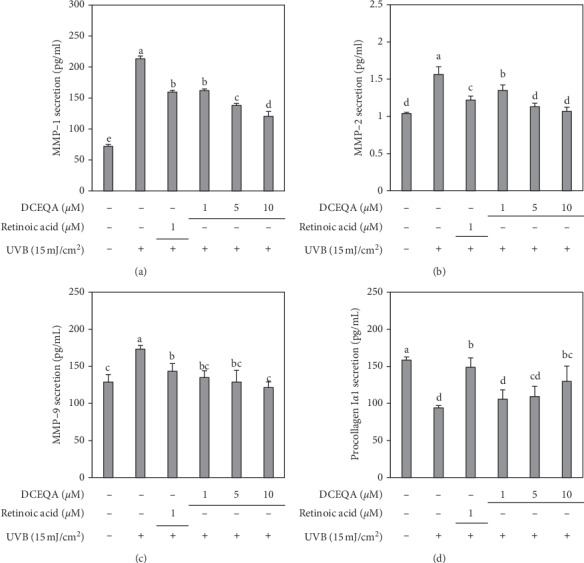
Effect of DCEQA on the secretion levels of MMP-1, MMP-2, MMP-9, and type Iα1 procollagen. Cells were treated with DCEQA at the indicated concentrations for 24 h following UVB irradiation (15 mJ/cm^2^). Released protein amounts from conditioned HaCaT cell culture medium were quantified by an ELISA kit. Values are mean ± SD of three independent experiments run in triplicate. ^a-e^Means with different letters are significantly different at *p* < 0.05 level.

Release of MMP-1, -2, and -9 and type I*α*1 procollagen from irradiated and nonirradiated HaCaT cells was analyzed by ELISA. UVB irradiation at a dose of 15 mJ/cm^2^ notably increased the amount of MMPs in the cell culture medium while resulting in a decreased amount of type I procollagen (Figure 3). Treatment of HaCaT keratinocytes with DCEQA (1, 5, and 10 *μ*M) after UVB exposure resulted in a dose-dependent decrease in the released amounts of MMP-1, -2, and -9. In addition, DCEQA treatment was able to increase the type I procollagen levels. DCEQA-mediated reduction of MMP release was comparable to the positive control retinoic acid (1 *μ*M). However, the increase in procollagen levels was still lower than that of retinoic acid treatment, though still significantly higher than the untreated control group.

**Figure 4:**
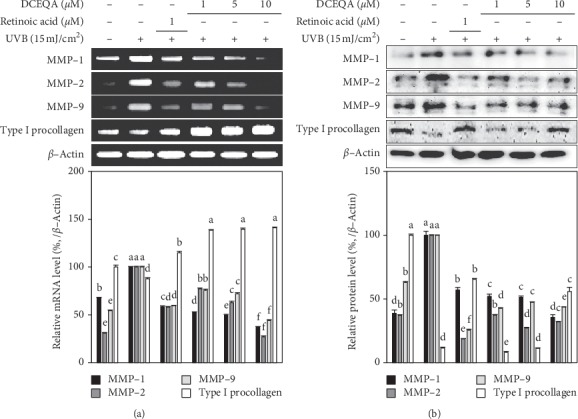
Effect of DCEQA on the mRNA and protein expression levels of the MMP-1, MMP-2, MMP-9, and type Iα1 procollagen in UVB-irradiated HaCaT keratinocytes after 24 h from treatment analyzed by RT-PCR and Western blotting, respectively. Expression levels were quantified using the density of the bands and normalized against the levels of housekeeping gene *β*-actin. The effect of DCEQA on expression levels was given as the relative percentage of the UVB-irradiated (15 mJ/cm^2^) untreated control cells. Values are mean ± SD of three independent experiments. ^a–f^Means with different letters are significantly different at *p* < 0.05 level.

Expectedly, UVB exposure at a dose of 15 mJ/cm^2^ caused overexpression of MMPs and a decrease in type I*α*1 procollagen synthesis in irradiated but untreated control cells. Treatment of cells with different concentrations of DCEQA after exposure to UVB radiation (15 mJ/cm^2^) ameliorated the overstimulation of MMP-1, -2, and -9 expression in both mRNA and protein levels (Figure 4). In addition, treatment with DCEQA increased the mRNA levels of type I procollagen to 141.10% of the nonirradiated and untreated blank group. Also, it was observed that DCEQA treatment showed a similar trend in type I procollagen protein levels, but lower than the increase seen in mRNA expression. Any discrepancies between the expression and cellular release levels were attributed to the toxic effects of UVB on HaCaT keratinocytes. Although all samples were subjected to normalization to test the same amounts of total proteins, apoptotic cells and their remains in supernatant could play a role in the cellular release levels. Nevertheless, considered together with the cytoprotective effects of DCEQA against UVB-induced cytotoxicity, these results suggested a potential MMP expression inhibitory activity for DCEQA.

### 3.4. Effect of DCEQA on MAPK Expression and Phosphorylation

**Figure 5:**
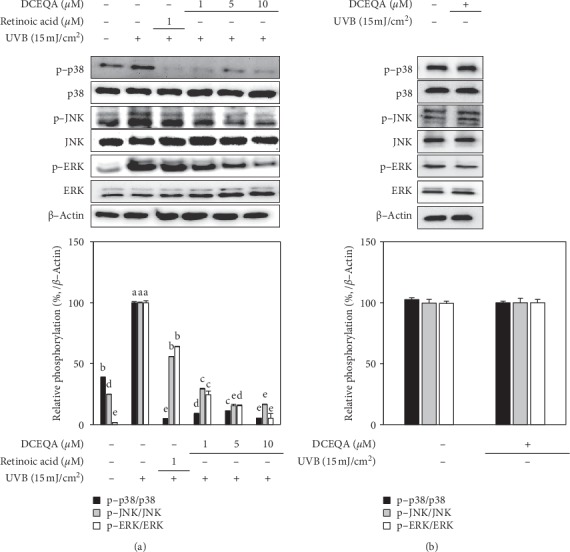
Effect of DCEQA on the protein expression levels of the inactive and phosphorylated (p-) p38, JNK, and ERK MAPKs in UVB-irradiated (a) and nonirradiated (b) HaCaT keratinocytes after 24 h from treatment, analyzed by Western blotting. Expression levels were quantified using the density of the bands and normalized against the levels of housekeeping gene *β*-actin. Phosphorylation levels were given calculating the relative fold change of phosphorylated protein to its inactive form as a percentage of UVB-irradiated (15 mJ/cm^2^) untreated control cells (a) or nonirradiated untreated control cells (b). Values are mean ± SD of three independent experiments. ^a–f^Means with different letters are significantly different at *p* < 0.05 level.

As seen in Figure 5(a), irradiation at a UVB dose of 15 mJ/cm^2^ significantly stimulated the phosphorylation of p38, ERK, and JNK MAPKs. After the introduction of DCEQA (1, 5, and 10 *μ*M), dose-dependent inhibition of p38, ERK, and JNK phosphorylation was detected (Figure 5(a)). The inhibitory effect of DCEQA on the phosphorylation of p38 and ERK was higher compared to JNK, and it was significant even at the lowest dose-treated (1 *μ*M). In addition, phosphorylation of p38 levels was suppressed to levels even lower than the nonirradiated, untreated blank group starting from the low doses. DCEQA-only treatment of the keratinocytes without UVB irradiation did not exert any significant changes in the phosphorylation of p38, ERK, and JNK MAPKs as seen in Figure 5(b). This suggested that the effect of DCEQA on the MAPK phosphorylation was UVB dependent.

**Figure 6:**
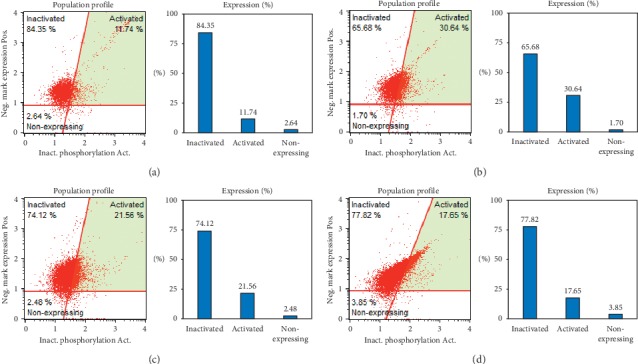
Effect of DCEQA on the activation of ERK MAPK in UVB-irradiated HaCaT keratinocytes after 24 h from treatment, analyzed by FACS flow cytometry. Plots give the percentage of cells with phosphorylated ERK in the total population. Blank: untreated nonirradiated cells; control: untreated UVB-irradiated cells. Retinoic acid was used as a positive control. (a) Blank, (b) control, (c) retinoic acid (1 *μ*m), (d) DCEQA (10 *μ*m).

The effect of DCEQA on the activation of ERK was further analyzed using fluorescence-activated cell sorting (FACS) flow cytometry. Exposure to UVB (15 mJ/cm^2^) increased the 11.74% activated ERK population in HaCaT keratinocytes to 30.64% following 24 h incubation (Figure 6). Treatment with DCEQA (10 *μ*M) dropped the cell population with activated ERK to 17.65%, while 1 *μ*M retinoic acid treatment as positive control decreased it to 21.56%.

## 4. Discussion

Exposure to UV radiation causes a series of complications in the skin. Skin cells, especially keratinocytes in the epidermis, demonstrated stimulated expression of MMPs due to elevated inflammation and ROS when they were irradiated with UVB [4]. This change in the regulation of MMPs results in the degradation of the ECM and the widely studied reason behind skin deformations known as photoaging [23]. Therefore, researches focused on developing MMP inhibitors as antiphotoaging agents to prevent the UVB-induced degradation of the skin collagen [24, 25]. Several studies reported very promising results indicating that ameliorating the MMP overexpression is a beneficial strategy against photoaging along with scavenging the UVB-induced ROS [26, 27]. Keratinocytes form the main defense mechanism of skin against UVB irradiation as UVB rays mainly affect epidermis layer. Hence, UVB-induced photoaging is initiated through the deterioration in keratinocytes. The current study, therefore, employed an in vitro immortalized keratinocyte model, HaCaT cells, in order to investigate potential properties of DCEQA against UVB-mediated photoaging. In a previous study, DCEQA was found to protect keratinocytes against UVB irradiation via regulation of antioxidant defense mechanism: scavenging the ROS and stimulating the antioxidant enzyme production via Nrf2 pathway [22]. The current study added more insight into the suggested antiphotoaging effect of DCEQA by showing that treatment with DCEQA not only protected cells from UVB-induced cytotoxicity but also suppressed MMP expression while increasing the type I procollagen content. Results indicated that DCEQA showed this effect via intervening with the phosphorylation of p38, JNK, and ERK MAPKs which was upregulated parallel to overexpression of MMPs following UVB irradiation.

Chronic exposure to UVB substantially enhances the activity of collagenases, hence, reduces the overall collagen production, and damages the collagen backbone of the extracellular matrix. This collagen backbone is crucial for the elasticity and strength of the skin, and any deterioration as a result of external stimuli results in prematurely aged skin or photoaged skin if the stimulus is UV radiation [4]. In this context, inhibition of the collagenases, MMPs, has been regarded as a potential therapeutic approach to prevent and treat photoaging, skin inflammation, and wrinkle formation [24]. Several studies reported natural products as promising antiphotoaging agents with strong oxidative stress quenching abilities due to their high polyphenol content [28, 29]. Most of the compounds isolated from these natural products showed strong antioxidant effect and inhibited the enzymatic activities of MMPs in various occasions. The ROS scavenging effect of DCEQA was previously reported against UVB-induced elevation of the oxidative stress in human keratinocytes [22]. Chiang et al. [30] showed that *Coffea arabica* extract and its major components, caffeic and chlorogenic acid, suppressed MMP expression via downregulation of MAPK pathway. Current results showed that DCEQA is a potent compound that could suppress the mRNA and protein expression of MMP-1, -2, and -9 and upregulate the production of type I procollagen (Figures 3 and 4). The presence of the DCEQA was able to strongly downregulate the phosphorylation of MAPKs as a suggested mechanism for its antiphotoaging action. Any suppression of the MAPK pathway results in decreased MMP activity and relieved collagen production to overcome the harmful effects of UVB irradiation [31, 32].

The activation of MAPK pathway is involved in several pathways in skin cells. UVB irradiation-induced photoaging is also suggested to manifest itself via MAPK activation. It was reported that the phosphorylation of MAPK proteins plays an important role in the production of MMPs in human dermal fibroblasts [32]. UVB-mediated elevation of ROS and other reactive species stimulates a set of signaling cascades ending with elevated inflammatory response and MAPK activation. Both mechanisms have been reported to affect collagen synthesis negatively. Studies showed that the suppression of UV-induced oxidative stress and MAPK activation not only attenuated MMP expression but also had beneficial effects on diminished collagen synthesis [33, 34]. Our previous study showed that DCEQA exhibited antiphotoaging properties via regulation of the oxidative stress defense mechanism as a prevention and/or treatment approach against UVB radiation since the harmful effects of UVB irradiation were mainly due to elevated ROS [22]. The present study showed that DCEQA was also able to suppress the MMP-1, -2, and -9 expression as a mechanism against UVB-induced photoaging by collagen degradation. In addition, DCEQA inhibited the p38, ERK, and JNK activation. The flow cytometry results (Figure 6) further suggested that DCEQA treatment specifically inhibited the ERK activation in UVB-irradiated HaCaT keratinocytes. Activation of these MAPKs is also closely linked with the synthesis and function of the AP-1 transcription factor [35]. AP-1 transcription factor, along with other factors such as c-Fos, is an important part of the MMP regulation and procollagen expression [36]. Based on the results and previous reports, it was speculated that DCEQA protects keratinocytes from UVB-induced photoaging. The action mechanism behind this bioactivity was suggested to involve regulation of intracellular antioxidant mechanism and amelioration of UVB-mediated deterioration of MMP expression via inhibition of MAPK activation.

## 5. Conclusion

UVB exposure causes serious skin complications such as inflammation, cancer, and photoaging. UVB-induced elevation of oxidative stress and overexpression of MMPs deteriorate the collagen composition of the skin. 3,5-Dicaffeoyl-epi-quinic acid attenuated UVB irradiation-induced overexpression of MMP-1, -2, and -9 and suppression of procollagen production in human HaCaT keratinocytes suggestively by inhibiting the stimulated MAPK activation. Overall, results indicated that DCEQA is a promising bioactive compound with a potential antiphotoaging effect in UVB-irradiated keratinocytes.

## Data Availability

All data used to support the findings of this study are available from the corresponding author upon reasonable request.
